# Prior cigarette smoke exposure does not affect acute post-stroke outcomes in mice

**DOI:** 10.1371/journal.pone.0214246

**Published:** 2019-03-21

**Authors:** Victoria Austin, Alyson Miller, Ross Vlahos

**Affiliations:** 1 School of Health and Biomedical Sciences, RMIT University, BUNDOORA, VIC, Australia; 2 Institute of Cardiovascular & Medical Sciences, Glasgow Cardiovascular Research Centre, University of Glasgow, Glasgow, Scotland, United Kingdom; Telethon Institute for Child Health Research, AUSTRALIA

## Abstract

Chronic obstructive pulmonary disease (COPD) is currently the third leading cause of death globally and is characterized by airflow limitation that is progressive and not fully reversible. Cigarette smoking is the major cause of COPD. Fifty percent of deaths in the COPD population are due to a cardiovascular event and it is now recognised that COPD is a risk factor for stroke. Whether COPD increases stroke severity has not been explored. The aim of this study was to investigate whether functional and histological endpoints of stroke outcomes in mice after transient middle cerebral artery occlusion (tMCAo) were more severe in mice exposed to cigarette smoke (CS). 7-week-old male C57BL/6 mice were exposed to room air or CS generated from 9 cigarettes/day, 5 days/week for 2, 8 and 12 weeks. Following air or CS exposure, mice underwent tMCAO surgery with an ischaemic period of 30–40 min or sham surgery. Mice were euthanised 24 h following the induction of ischaemia and bronchoalveolar lavage fluid (BALF), lungs and brains collected. Mice exposed to CS for 2 weeks and subjected to a stroke had similar BALF macrophages to air-exposed and stroke mice. However, CS plus stroke mice had significantly more BALF total cells, neutrophils and lymphocytes than air plus stroke mice. Mice exposed to CS for 8 and 12 weeks had significantly greater BALF total cells, macrophages, neutrophils and lymphocytes than air-exposed mice, but stroke did not affect CS-induced BALF cellularity. Prior CS exposure did not worsen stroke-induced neurological deficit scores, reduced foregrip strength, infarct and oedema volumes. Collectively, we found that although CS exposure caused significant BALF inflammation, it did not worsen acute post-stroke outcomes in mice. This data suggests that while patients with COPD are at increased risk of stroke, it may not translate to COPD patients having more severe stroke outcomes.

## Introduction

Chronic obstructive pulmonary disease (COPD) is currently the third leading cause of death globally [1, 2]. Cigarette smoking is the major cause of COPD and accounts for more than 95% of cases in industrialized countries [3], but other environmental pollutants are important causes particularly in developing countries [4]. COPD is characterized by an airway limitation that is usually progressive and not fully reversible [5], and is associated with a chronic and abnormal inflammatory response in the airways in response to noxious gases and particles [1]. COPD encompasses chronic obstructive bronchiolitis with fibrosis and obstruction of small airways, and emphysema with enlargement of airspaces and destruction of lung parenchyma, loss of lung elasticity, and closure of small airways. Most patients with COPD have all three pathological conditions (chronic obstructive bronchiolitis, emphysema and mucus plugging), but the relative extent of emphysema and obstructive bronchiolitis within individual patients.

Several inflammatory cell types are involved in the pathophysiology of COPD, including macrophages, neutrophils and T-cells [6, 7]. These inflammatory cells have an impaired phagocytic function, resulting in impairment in clearance of apoptotic cells, contributing to the chronic inflammatory state in the lungs and leading to an ongoing cycle of damage and remodelling in the airways and lung tissue [8, 9]. In addition to local inflammation in the lungs, COPD is associated with chronic systemic inflammation and oxidative stress [10–14]. This state of chronic systemic inflammation is believed to be involved in the development of comorbidities of COPD [15, 16].

Much of the disease burden of COPD is associated with the management of comorbidities, rather than the airway limitation itself. It is estimated that between 30–50% of deaths in the COPD population are due to cardiovascular events including myocardial infarction [17–19]. In addition to this, COPD is increasingly being recognized as a risk factor for stroke [20]. Recent studies show that the risk of stroke is 20% greater in COPD patients compared to the general population, and this risk is estimated to be 7-fold higher following an exacerbation of COPD [21, 22]. There is also some evidence to suggest that there is a relationship between COPD and worse stroke outcomes such as mortality, pneumonia, epilepsy, length of hospital stay and also intensive care unit care [23]. COPD is an independent risk factor for mortality among patients with stroke [24], and stroke patients with COPD are at an increased risk of aspiration [25], which is a leading cause of death following a stroke.

However, no causal mechanism has been established between COPD and worse outcomes following a stroke. Heightened levels of systemic inflammation and oxidative stress may contribute to increased stroke severity and the occurrence of post-stroke adverse events [20, 23]. It is difficult to determine the causal mechanisms that may explain worse outcomes following a stroke in human COPD. One way to potentially understand the mechanistic links between cigarette smoke-induced COPD and stroke outcomes is to use an animal model that combines cigarette smoke and stroke. Therefore, the aim of this study was to investigate whether prior cigarette smoke exposure worsens brain injury and stroke outcomes (functional hanging wire test, neurological scoring, infarct and oedema volume) in mice.

## Materials & methods

### Mice

All experiments were conducted in accordance with the Australia Code of Practice for the Care of Experimental Animals, the ARRIVE Guidelines and with RMIT University Animal Ethics Committee approval (Animal Ethics Application Number 1532). Male 7-12-week-old C57BL/6 mice (n = 147) were obtained from the Animal Resources Centre (Perth, Australia). Animals were housed on a 12 h light/dark cycle and had access to water and standard chow ad libitum. In total, 35 mice were excluded from the study which occurred when, during the surgical procedure: (1) there was an inadequate reduction (<70%) in regional cerebral blood flow (rCBF) during the ischemic period or inadequate (>80%) increase within the first 10 minutes of reperfusion (n = 23); (2) technical or anaesthesia complications arose during surgery (n = 3); (3) they died prior to the end of the reperfusion period (n = 7); or (4) they had to be humanely killed due to severity of adverse effects of stroke (n = 2).

### Cigarette smoke exposure

Mice were randomly assigned to either sham (room air) or cigarette smoke (CS) exposure groups. Mice were then placed in an 18-L perspex chamber (The Plastic Man, Huntingdale, Victoria, Australia) in a standard chemical hood and exposed to CS generated from 9 cigarettes per day, 5 days per week for 2 (acute exposure), 8 (sub-chronic exposure) and 12 (chronic exposure) weeks as previously described [26]. Briefly, mice were exposed to CS generated from 9 cigarettes/day for 2, 8 and 12 weeks (Monday to Friday but not Saturday and Sunday), delivered three times per day at 9 AM, 12 PM and 3 PM with 3 cigarettes spaced over 1 h. CS was generated in 50-ml tidal volumes over 10 s, by use of timed draw-back mimicking normal smoking inhalation volume and cigarette burn rate. The mean total suspended particulate mass concentration in the chamber containing CS was ~420 mg m^-3^ [26]. Commercially available filter-tipped Winfield Red cigarettes (manufactured by Philip Morris, Australia) of the following composition were used: 16 mg or less of tar, 1.2 mg or less of nicotine, and 15 mg or less of CO. Sham-exposed mice were placed in an 18-L perspex chamber but were not exposed to CS. The acute cigarette smoke-exposure protocol was used as it typically explores the mediators and mechanisms involved in the induction of cigarette smoke-induced lung inflammation and damage. The sub-chronic cigarette smoke-exposure protocol was used as it typically explores the mediators and mechanisms involved in the progression of cigarette smoke-induced lung inflammation and damage. We have also shown that mice exposed to 8 weeks of cigarette smoke have lung inflammation, changes in lung function and structure and extrapulmonary manifestations including skeletal muscle wasting and dysfunction (unpublished observations). The chronic protocol was used as it typically causes more severe lung inflammation and pathology (e.g. emphysema, small airway fibrosis). Thus, these exposure protocols provide a robust clinically-relevant platform to explore mechanisms that are relevant to cigarette smoke-induced COPD and its comorbidities.

### Focal cerebral ischemia and reperfusion

Mice were anaesthetised with a mixture of ketamine (150 mg/kg, i.p.) and xylazine (10 mg/kg, i.p.). Body temperature was maintained at 37°C with a heat lamp throughout the procedure and until mice regained consciousness. Mice were kept on a heat-pad post-operatively. Focal cerebral ischemia and reperfusion was performed on mice by transient intraluminal filament-induced middle cerebral artery occlusion (tMCAo) as previously described [27–29]. Cerebral ischemia was maintained for 30 min (2-week CS exposure) or 40 min (8 and 12-week CS exposure). Given that we thought CS exposure would significantly exacerbate infarct volume, an ischemic period of 30 min was used for the 2-week CS exposure protocol to induce a small infarct such that an exacerbated response would not significantly affect the well-being of the mice or lead to increased mortality. rCBF in the area of the cortex supplied by the middle cerebral artery (MCA) (~2 mm posterior and 5 mm lateral to bregma) was monitored in all stroke mice and recorded prior to the induction of cerebral ischemia, during cerebral ischemia and for the first 10 min of reperfusion. For sham surgeries (8 and 12-week CS protocol), the right external carotid artery and common carotid artery were visualised but the filament was not inserted. After mice had recovered from anaesthesia, they were housed in individual cages. Mice were monitored hourly for a minimum of 8 h post-surgery and the following morning using our monitoring protocol and clinical signs severity scoring system (approved by our ethics committee). Mice were killed 24 hours post-surgery with an overdose of isoflurane followed by decapitation to determine whether cigarette smoke exposure impacts acute post-stroke outcomes. No sham surgeries were performed for the 2-week CS exposure group as our initial primary goal was to see whether CS-exposure exacerbated stroke outcomes, to ensure that mice would recover from stroke surgeries after CS exposure and to reduce animal usage.

### Neurological scoring and functional impairment test

Neurological assessment was performed 24 h after either sham or stroke surgery using a five-point scoring system: 0 = normal motor function; 1 = flexion of torso and contralateral forelimb when lifted by the tail; 2 = circling to the contralateral side when held by the tail on a flat surface with normal posture at rest; 3 = leaning on the contralateral side at rest; 4 = no spontaneous movement at rest or uncontrolled circling. A hanging wire test was performed at 24 h after sham or stroke surgery to assess motor impairment, as previously described [27]. Briefly, mice were suspended by their forelimbs from a wire 30 cm above a padded surface for up to 60 s and the average hanging time (i.e. latency to fall) of 3 trials with 5 min rest in between was recorded. A score of zero was assigned to those mice that fell immediately and a score of 60 was assigned to animals that did not fall.

### Quantification of cerebral infarct and oedema volumes

Cerebral infarct and oedema volumes were evaluated as previously described [27]. Briefly, brains were coronally sectioned (30 μm thickness; 420 μm apart) and thaw mounted onto 0.1% poly-L-lysine coated slides. Tissue-mounted slides were subsequently stained with 0.1% thionin to delineate the infarct. Thionin-stained sections were then imaged with an Olympus VS120 Slide Scanner (Olympus). Total infarct volume was then quantified using ImageJ image analysis software, correcting for brain oedema, as previously described [27].

### Bronchoalveolar lavage and differential cell counts

Lungs were lavaged in situ with a 400 μl aliquot of PBS, followed by three 300 μl aliquots as previously described [26, 30, 31]. In total up to 1 ml of bronchoalvealor lavage fluid (BALF) was retrieved per mouse. The total number of viable cells in the BALF was determined using the fluorophores ethidium bromide and acridine orange (AO/EB), on a Nikon Eclipse E600 (Nikon Instruments, USA). Cytospins were prepared using 100 μl of BALF spun at 400 rpm for 10 min using a Cytospin 3 (Shandon, UK). Cytospin preparations were then stained with DiffQuik (Dade Baxter, Australia), and 500 cells per slide were counted and differentiated into macrophages, neutrophils and lymphocytes using standard morphological criteria.

### RNA extraction and qPCR

Lungs from individual mice were crushed to a fine powder in liquid nitrogen using a mortar and pestle, and subsequently 15 mg homogenised by passing 5 times through a 21G needle with a 1 ml syringe. Total RNA was extracted from lung samples using an RNeasy Plus kit (QIAGEN, Australia), according to the manufacturer’s instructions. RNA yield and purity were quantified using a nanodrop (ND-1000, Biolab). Total RNA from lung samples were reverse transcribed to cDNA (Applied Biosystems High Capacity RNA-to-cDNA Kit, USA). Quantitative polymerase chain reaction (qPCR) was then performed using mouse-specific TaqMan Gene Expression Assays (Applied Biosystems, USA), on an ABI 7900HT Sequence Detection System. Samples were assayed in triplicate and negative reverse-transcriptase controls were included. Fold change was determined relative to the sham control group, after standardising to GAPDH (housekeeping gene), using the standard 2^(-ΔΔCT)^ method as previously published [26, 30, 32, 33].

### Data analysis

All results are presented as mean±standard error of the mean (SEM); n represents the number of mice. Statistical comparisons between treatment groups were performed using either Student’s unpaired t test or two-way ANOVA with Bonferroni multiple comparisons post-hoc test. Mann-Whitney U test was used for non-parametric data. All statistical analyses were performed using GraphPad Prism 6 for Windows (Version 6.07, La Jolla, CA, USA). Probability levels less than 0.05 (P<0.05) were taken to indicate statistical significance.

## Results

### Effect of cigarette smoke exposure on body weight and lung inflammation

**Fig 1:**
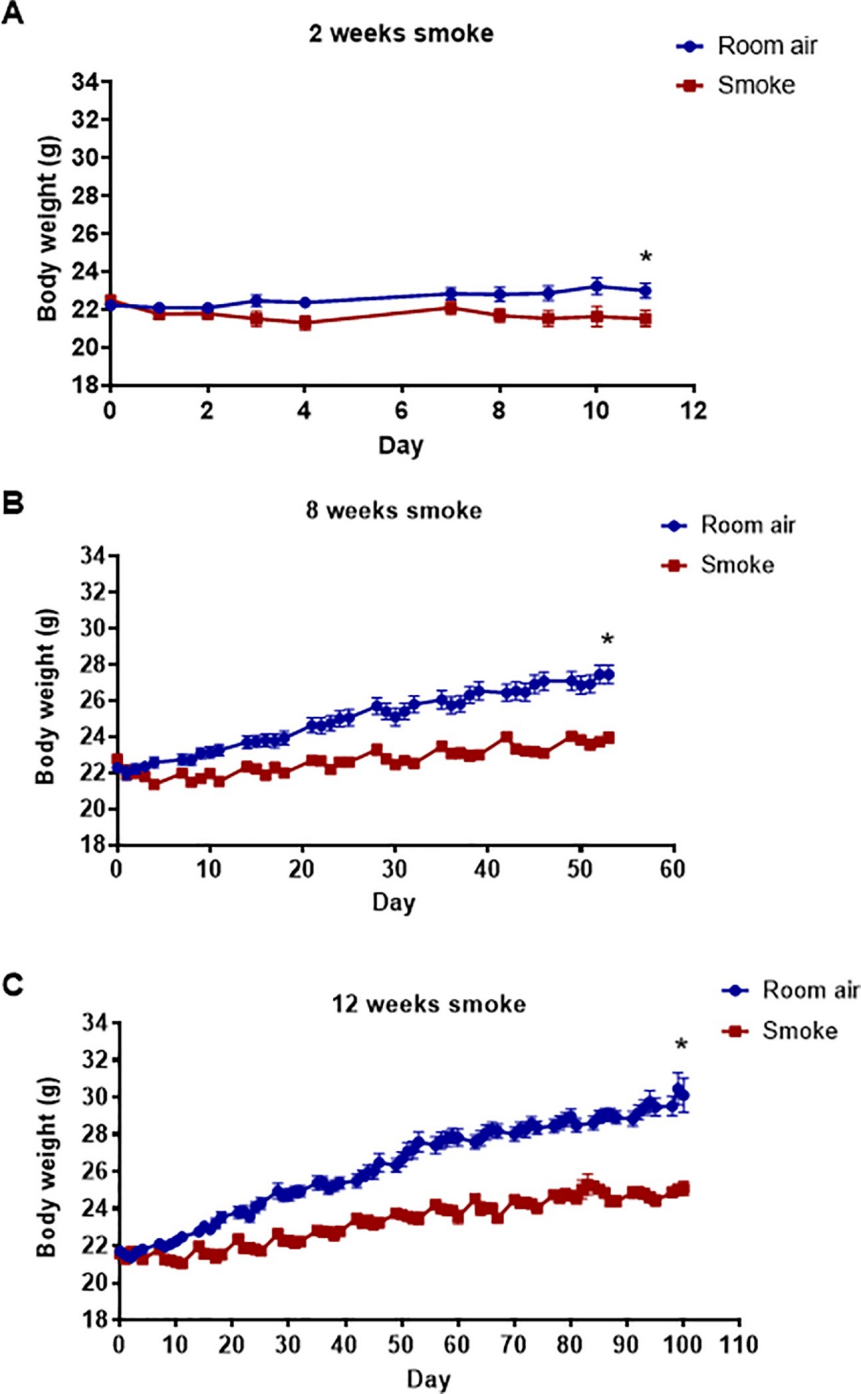
Effect of cigarette smoke exposure on body weight. Mice were exposed to cigarette smoke or room air (sham) for 2 weeks (A; n = 10), 8 weeks (B; n = 18) or 12 weeks (C; n = 35–47). Data are expressed as mean ± SEM (Student’s unpaired t-test performed on final weights, *P<0.05 vs room air-exposed group).

We first examined the effect of acute (2 weeks), sub-chronic (8 weeks) and chronic (12 weeks) CS exposure on body weight. The body weight of mice exposed to CS for 2, 8 and 12 weeks was lower than mice exposed to room air for these timepoints (Fig 1 and S1 Fig).

**Fig 2:**
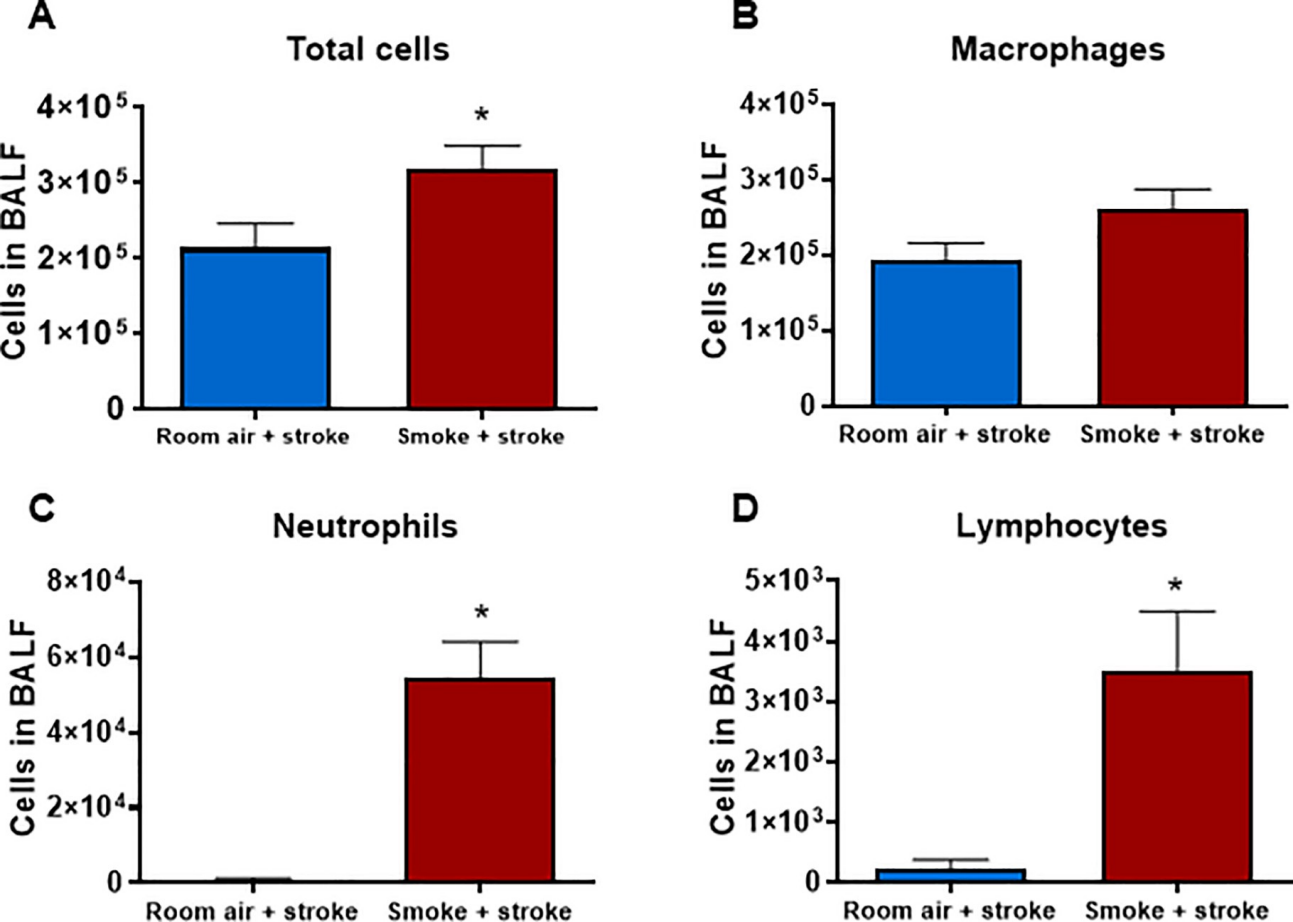
Effect of 2 weeks cigarette smoke exposure on inflammatory cells in bronchoalveolar lavage fluid (BALF). Total (A), macrophage (B), neutrophil (C) and lymphocyte (D) cell counts shown as mean ± SEM (n = 5–6, *P<0.05 vs room air stroke mice, Student’s unpaired t-test).

We then went on to determine whether stroke and CS exposure influenced BALF cellularity after 2, 8 and 12 weeks of CS exposure. Mice exposed to CS and for 2 weeks and subjected to a stroke had similar BALF macrophages to room air exposed and stroke mice (Fig 2). However, CS plus stroke mice had significantly more BALF total cells, neutrophils and lymphocytes than room air plus stroke mice (Fig 2, *P*<0.05, Student’s unpaired t-test; S2 Fig).

**Fig 3:**
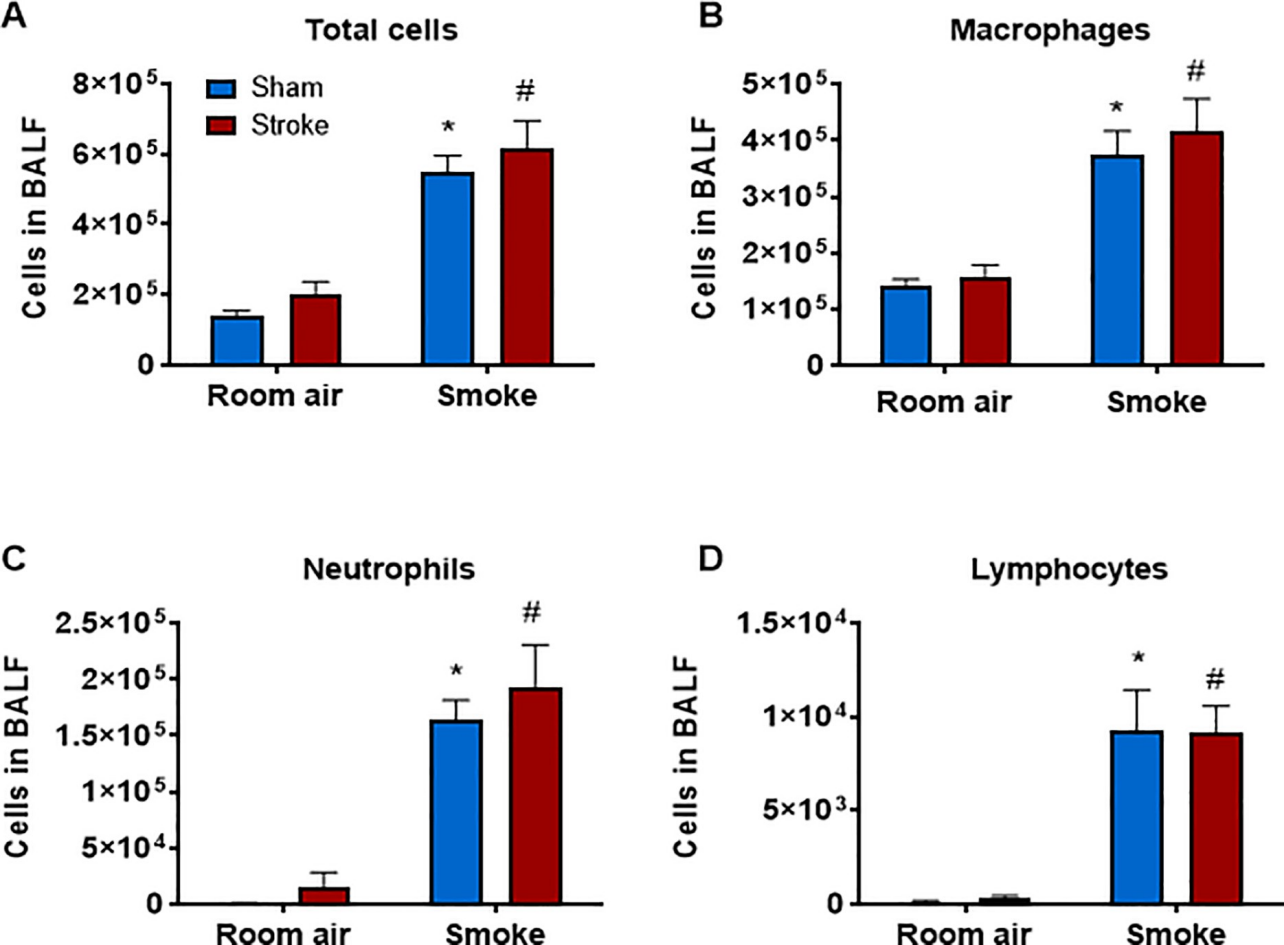
Effect of 8 weeks of cigarette smoke exposure on inflammatory cell counts in bronchoalveolar lavage fluid (BALF). Total (A), macrophage (B), neutrophil (C) and lymphocyte (D) cell counts shown as mean ± SEM (n = 5–7, *P<0.05 vs room air group, #*P***<**0.05 vs room air stroke group, two-way ANOVA followed by Bonferroni post-hoc test).

**Fig 4:**
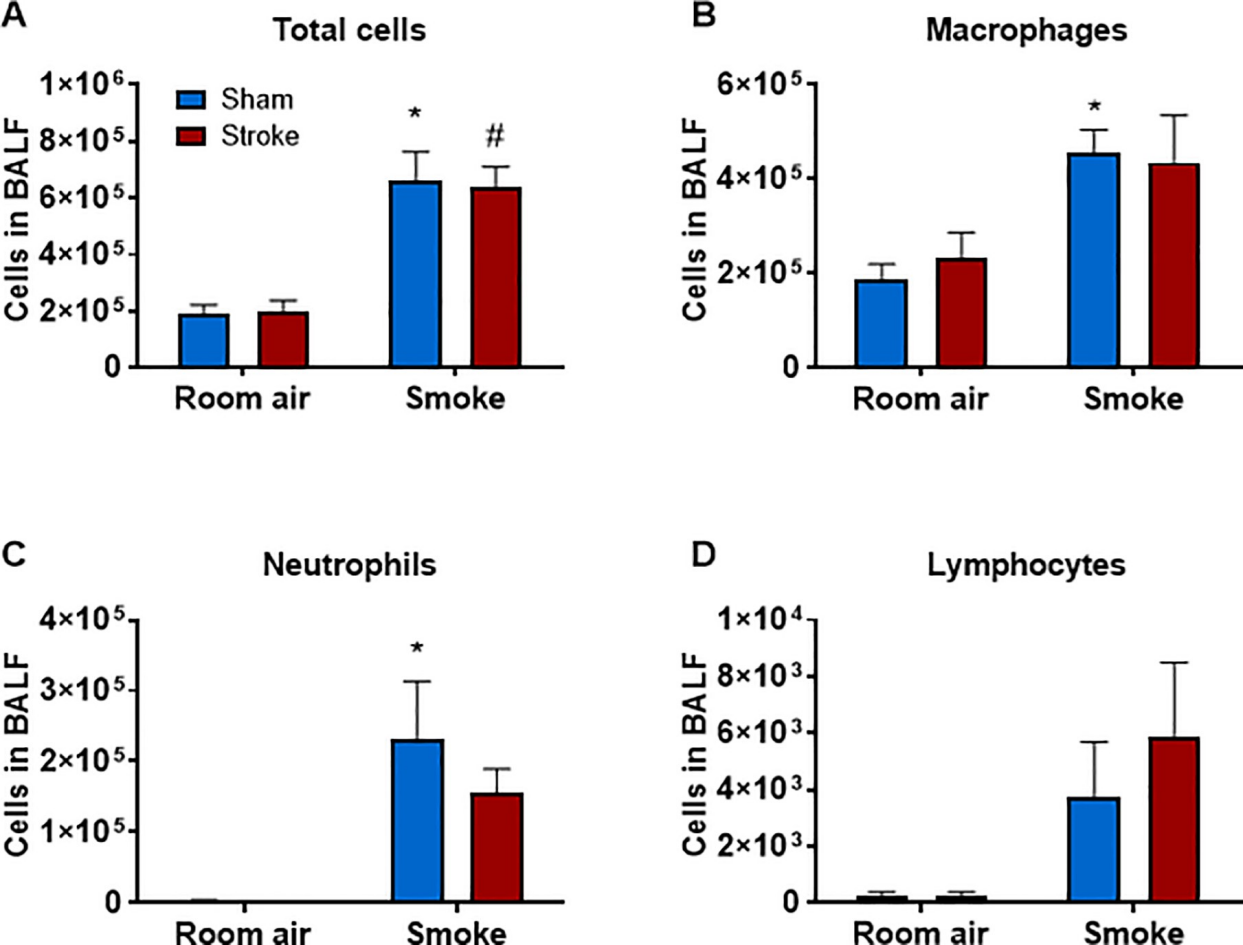
Effect of 12 weeks cigarette smoke exposure on inflammatory cell counts in bronchoalveolar lavage fluid (BALF). Total (A), macrophage (B), neutrophil (C) and lymphocyte (D) cell counts shown as mean ± SEM (n = 4–8, *P**<**0.05 vs room air sham group, #*P***<**0.05 vs room air stroke group, two-way ANOVA followed by Bonferroni post-hoc test).

Mice exposed to CS for 8 weeks had significantly greater BALF total cells, macrophages, neutrophils and lymphocytes (Fig 3 and S3 Fig) than air-exposed mice (*P*<0.05, two-way ANOVA followed by Bonferroni post-hoc test). Stroke alone did not increase BALF cellularity in room air-exposed mice nor did it affect CS-induced BALF cellularity. Similarly, mice exposed to CS for 12 weeks had significantly greater BALF total cells, macrophages and neutrophils (but not lymphocytes) than air-exposed mice (*P*<0.05, two-way ANOVA followed by Bonferroni post-hoc test) (Fig 4 and S4 Fig). Stroke alone did not increase BALF cellularity in room air-exposed mice nor did it affect CS-induced BALF cellularity.

### Degree of hypoperfusion following stroke

**Fig 5:**
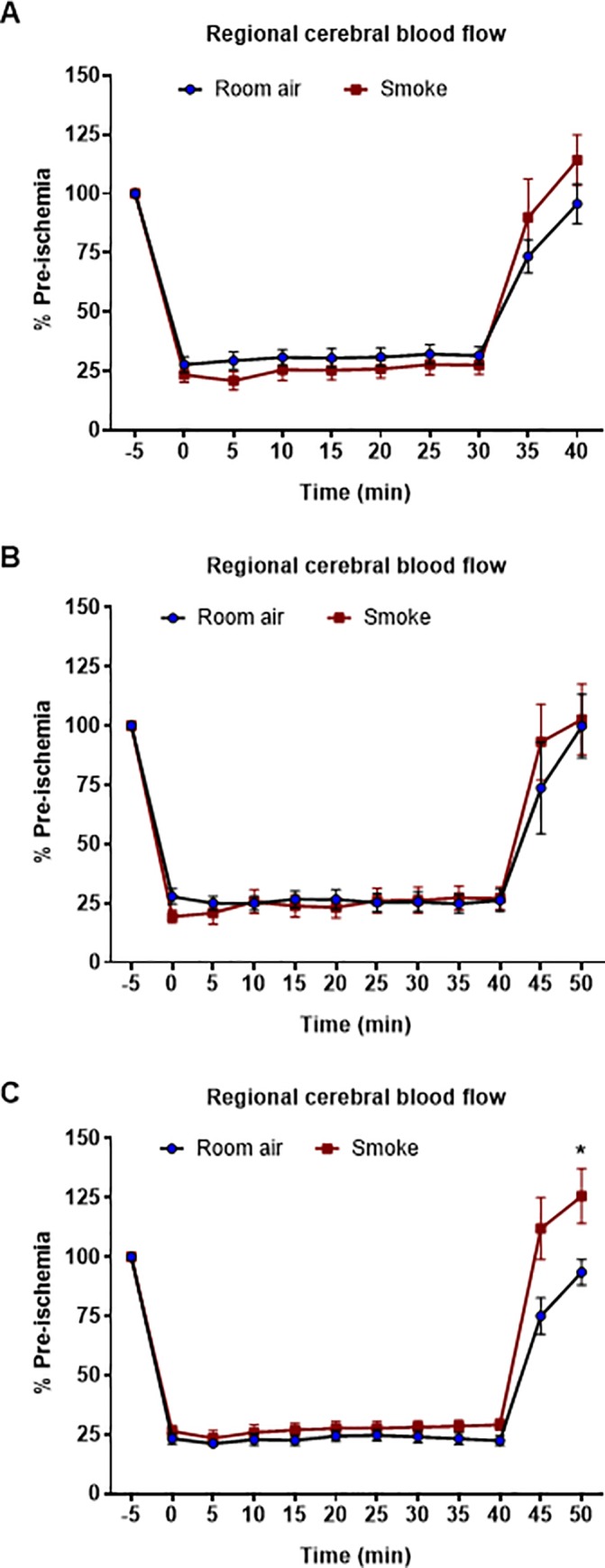
Regional cerebral blood flow during tMCAO surgery and during reperfusion. Surgeries were performed after 2 weeks (A; n = 8–10), 8 weeks, (B; n = 5–7) and 12 weeks (C; n = 8–10). Student’s unpaired t-test performed on final values, *P<0.05 vs room air group.

Following insertion of the monofilament at the origin of the MCA, all stroke mice experienced a similar drop (~75%) in rCBF (Fig 5 and S5 Fig) and this remained steady over the ischaemic period for all groups. A similar degree of reperfusion was observed in all mice in acute (2 weeks), sub-chronic (8 weeks) and chronic (12 week) experiments, though CBF during reperfusion was greater in the 12 weeks CS-exposed mice relative to room-air mice (*P*<0.05, Student’s unpaired t-test; Fig 5C).

### Effect of cigarette smoke exposure on stroke outcomes

**Fig 6:**
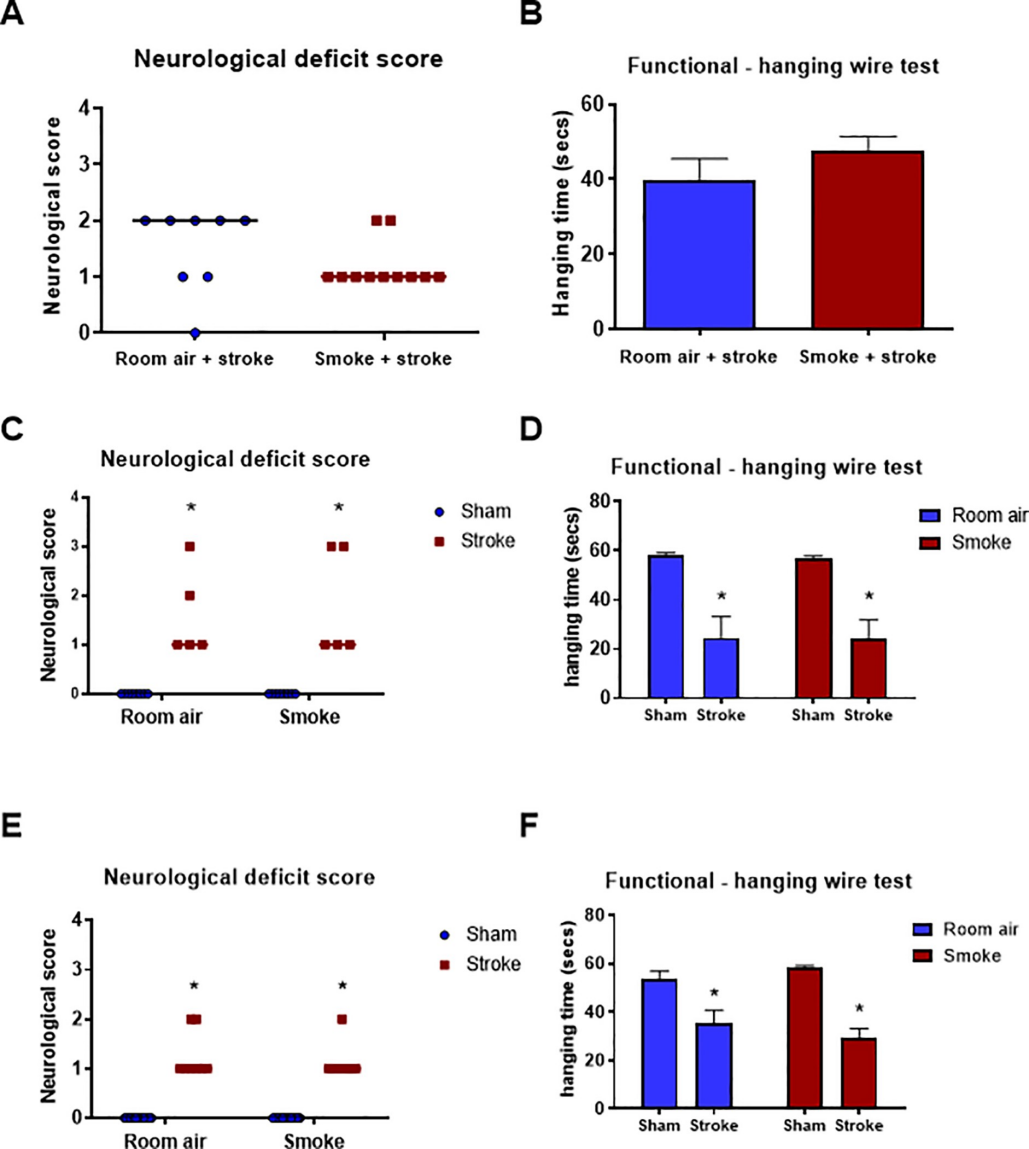
Neurological deficit and functional hanging wire scores 24 h post-stroke or sham surgery. Mice were exposed to 2 (A&B; n = 8–11), 8 (C&D; n = 5–8) or 12 (E&F; n = 8–18) weeks of cigarette smoke or sham smoke (room air) prior to sham or stroke surgery. Results for neurological scores are presented as median (line) and analysed with non-parametric Mann-Whitney U test (*P<0.05 vs sham). Results for hanging wire test are expressed as mean ± SEM. (*P<0.05 vs. sham, two-way ANOVA followed by Bonferroni post-hoc test).

All stroke animals displayed some degree of neurological deficit 24 h after stroke (Fig 6 and S6 Fig). Mice exposed to CS for 2 weeks and subjected to a stroke had similar neurological deficit and foregrip strength, as assessed by latency to fall in the hanging wire test, to room air-exposed and stroke mice (Fig 6A and 6B). Neurological deficit scores and foregrip strength in experimental stroke groups were significantly different from sham surgery control groups in the sub-chronic (8 weeks) and chronic (12 weeks) room air and CS exposure groups (Fig 6C, 6D, 6E and 6F; *P*<0.05, two-way ANOVA followed by Bonferroni *post-hoc* test or Mann-Whitney U test). However, prior CS exposure (acute, sub-chronic and chronic) did not worsen stroke-induced neurological deficit scores and reduced foregrip strength. All stroke groups had a significant reduction in hanging time on the hanging wire test, and this was significantly different from sham surgery control groups in the sub-chronic and chronic smoke exposure studies (Fig 6C, 6D, 6E and 6F; *P*<0.05, two-way ANOVA followed by Bonferroni post-hoc test).

### Effect of cigarette smoke exposure on infarct and oedema

**Fig 7:**
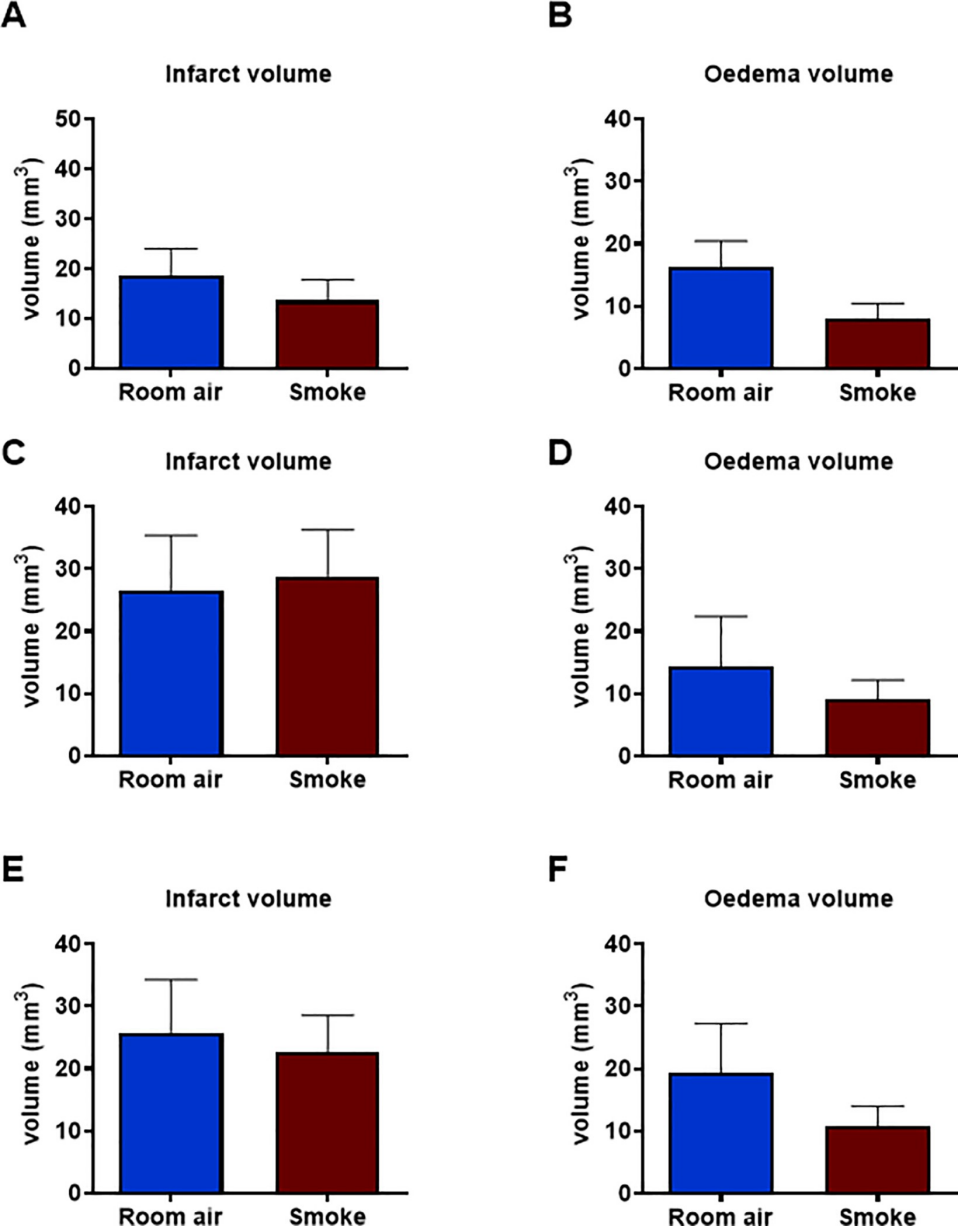
Cerebral infarct and oedema volumes at 24 h after tMCAO procedure. Mice were exposed to 2 (A&B; n = 5–10), 8 (C&D; n = 5) or 12 (E&F; n = 6–8) weeks of cigarette smoke or room air (sham) prior to stroke surgery. All results are expressed as mean ± SEM.

We then went on to investigate whether prior CS exposure influenced infarct and oedema volumes in stroke mice. No statistical differences in infarct volume were observed between smoke and sham groups after 2, 8 or 12 weeks of CS exposure (Fig 7 and S7 Fig). However, there was a trend for oedema volumes to be lower in CS-exposed animals compared to room air-exposed controls, but this was not statistically significant (*P*>0.05).

## Discussion

This study investigated whether prior cigarette smoke exposure worsens brain injury and stroke outcomes (functional hanging wire test, neurological scoring, infarct and oedema volume) in mice. The cigarette smoke exposure protocols used in this study replicate many aspects of human COPD and have been used previously to investigate the molecular and biochemical mechanisms underlying the pathogenesis of COPD [26, 34]. Briefly, the model involves exposing mice to CS over a period of days to months, depending on what features of cigarette smoke-induced lung inflammation and damage want to be modelled. Mice begin to develop an inflammatory response to CS after 4 days of CS exposure and begin to develop emphysema and a lung phenotype comparable to COPD after 3–6 months of exposure to CS. This model typically uses BALB/c mice, as they have a robust response to the CS [26]. However, in this study we used C57BL/6 mice, as their response to tMCAo is well established [27, 29]. In addition, we have shown in preliminary studies that BALB/c mice have a very high mortality rate, so it is not ethical to use the BALB/c strain of mouse for these studies.

It was hypothesized that chronic CS exposure would lead to worse stroke outcomes in mice. CS exposure causes an immediate inflammatory response in the lungs, by triggering the activation of proinflammatory mediators such as TNF-α NFκB and MMP-12, resulting in the recruitment of inflammatory cells such as neutrophils, macrophages and lymphocytes into the lungs. This immune response contributes to the damage of lung tissue [26]. After 2, 8 and 12 weeks of CS exposure inflammatory cells were elevated in the BALF, indicating that that lung inflammation was present in these mice. However, these cell counts after 2 weeks of CS exposure were lower than what has historically been seen in the BALB/c mouse strain in response to CS after 4 days [26, 31, 35]. We have previously shown that the C57BL/6 mouse strain is more resistant to acute CS-induced lung inflammation compared to the BALB/c mouse strain [26]. Considering this, we extended the CS exposure period out to 8 (sub-chronic protocol) and 12 (chronic protocol) weeks. CS exposure led to a reduction in body weight across all three time-points (2, 8 and 12 weeks). Nicotine, a primary constituent of CS, is known to suppress appetite. Although food intake was not measured in this study, we have previously shown that CS-exposed mice eat less compared to sham-exposed control mice [36]. However, studies have shown that the weight loss in response to CS is not solely due to a reduction in appetite, but that other metabolic pathways are also involved, such as decreases in energy intake, increases in energy expenditure and accelerated proteolysis [37, 38].

It was found that although CS exposure caused its expected effects on body weight (i.e. reduced body weight) and lung inflammation (i.e. increase in lung inflammation), there was no effect on stroke outcomes measured by neurological scoring, functional tests and analysis of infarct and oedema volumes in brain tissue. This was a surprise, given that there is increasing evidence to suggest that COPD is associated with worse outcomes (e.g. mortality, pneumonia, epilepsy, length of hospital stay, intensive care unit care) following stroke in humans [24]. It has been suggested that this may be due to increased systemic inflammation and oxidative stress making the brain more susceptible to brain injury, and the whole body more susceptible to adverse events following a stroke [20]. The results of this study suggest that the mouse model of CS-induced lung inflammation and damage used (i.e. 2, 8 and 12 weeks of CS exposure) has no effect on stroke severity. There are a number of possible reasons why stroke severity was not worse in this study: (i) 12 weeks of CS exposure may have been insufficient to induce systemic changes believed to play a key role in worsened stroke outcomes, (ii) COPD may not directly lead to worsened stroke severity, but may make an individual more susceptible to post-stroke complications and adverse events, and (iii) COPD may increase risk but play no role in stroke severity. Another important point to consider is that all mice in this study were culled 24 h post-stroke. In rodents, the vast majority (∼70–80%) of the infarct volume development takes place during the first 24 h [39]. However, we cannot rule out that CS-exposure may influence absolute final infarct volume [39]. Therefore, to assess long-term outcomes after stroke, future experiments should compare stroke outcomes in the days and weeks following experimental stroke in COPD mice and non-COPD mice. This will allow us to determine if COPD is causally linked to stroke severity and increased mortality and if so, to investigate the underlying mechanisms. Future experiments will also require an assessment of the systemic inflammation and oxidative stress in C57BL/6 mice after CS exposure. The CS exposure period may need to be lengthened for C57BL/6 mice, as they are not as sensitive to CS-induced inflammation as the BALB/c mouse strain, and systemic changes may not be occurring after 12 weeks of cigarette smoke exposure. Finally, it is possible that COPD may not impact on stroke severity but may just increase the risk of stroke. The model of stroke used in this study cannot be used to investigate risk; however, it can be used to investigate mechanisms and physiological changes that would likely increase the risk of a stroke. It is known that CS increases the risk of stroke through a number of mechanisms, such as hypercoagulability and increased immunoreactivity in atherosclerotic plaques. These changes may be sustained in COPD. Future studies will investigate cardiovascular changes in this preclinical animal model of COPD, which may elicit an increased risk of stroke.

In conclusion, this study found that although CS exposure caused significant BALF inflammation, it did not worsen acute post-stroke outcomes in mice measured by neurological scoring, functional tests and analysis of infarct and oedema volumes in brain tissue. Future studies should investigate stroke outcomes in the weeks following a stroke, and the effect of CS exposure on stroke risk.

## Supporting information

S1 Fig Bodyweight raw data. Mice were exposed to cigarette smoke or room air (sham) for 2, 8 or 12 weeks.
(PZFX)

S2 Fig BALF cell count raw data. The effect of 2 weeks cigarette smoke exposure on inflammatory cells in BALF.
(PZFX)

S3 Fig BALF cell count raw data. The effect of 8 weeks cigarette smoke exposure on inflammatory cells in BALF.
(PZFX)

S4 Fig BALF cell count raw data. The effect of 12 weeks cigarette smoke exposure on inflammatory cells in BALF.
(PZFX)

S5 Fig rCBF raw data. Surgeries were performed after 2, 8 and 12 weeks of cigarette smoke exposure.
(PZFX)

S6 Fig Neurological deficit and functional hanging wire raw data scores. Mice were exposed to 2, 8 or 12 weeks of cigarette smoke or sham smoke (room air) prior to sham or stroke surgery.
(PZFX)

S7 Fig Cerebral infarct and oedema volume raw data. Mice were exposed to 2, 8 or 12 weeks of cigarette smoke or room air (sham) prior to stroke surgery.
(PZFX)
